# Harnessing a Transient Gene Expression System in *Nicotiana benthamiana* to Explore Plant Agrochemical Transporters

**DOI:** 10.3390/plants10030524

**Published:** 2021-03-11

**Authors:** Bingqi Wu, Zhiting Chen, Xiaohui Xu, Ronghua Chen, Siwei Wang, Hanhong Xu, Fei Lin

**Affiliations:** 1 State Key Laboratory for Conservation and Utilization of Subtropical Agro-Bioresources/Key Laboratory of Natural Pesticide and Chemical Biology, Ministry of Education, South China Agricultural University, Guangzhou 510642, China; bingqiwu@stu.scau.edu.cn (B.W.); chenzhiting@gdaas.cn (Z.C.); 20186138006@stu.scau.edu.cn (X.X.); ronghuachen@stu.scau.edu.cn (R.C.); 1950774023@stu.scau.edu.cn (S.W.); 2 Institute of Quality Standard and Monitoring Technology for Agro-Products of Guangdong Academy of Agricultural Sciences, Guangzhou 510642, China; 3 Guangdong Province Key Laboratory of Microbial Signals and Disease Control, South China Agricultural University, Guangzhou 510642, China

**Keywords:** pesticide transporter, uptake, tobacco transient gene expression system, nicotine, neonicotinoid, pesticides

## Abstract

Functional characterization of plant agrichemical transporters provided an opportunity to discover molecules that have a high mobility in plants and have the potential to increase the amount of pesticides reaching damage sites. *Agrobacterium*-mediated transient expression in tobacco is simple and fast, and its protein expression efficiency is high; this system is generally used to mediate heterologous gene expression. In this article, transient expression of tobacco nicotine uptake permease (*NtNUP1*) and rice polyamine uptake transporter 1 (*OsPUT1*) in *Nicotiana benthamiana* was performed to investigate whether this system is useful as a platform for studying the interactions between plant transporters and pesticides. The results showed that *NtNUP1* increases nicotine uptake in *N. benthamiana* foliar discs and protoplasts, indicating that this transient gene expression system is feasible for studying gene function. Moreover, yeast expression of *OsPUT1* apparently increases methomyl uptake. Overall, this method of constructing a transient gene expression system is useful for improving the efficiency of analyzing the functions of plant heterologous transporter-encoding genes and revealed that this system can be further used to study the functions of transporters and pesticides, especially their interactions.

## 1. Introduction

Both the uptake and transport of agrochemicals are important factors influencing whether agrochemicals reach sites of pest damage and subsequently exert their activity. Agrochemicals must pass through the barrier of the plant plasma membrane before they arrive at the vascular systems for long-distance transport [1]. Transporter-mediated agrochemical transport provides a new approach to increase agrochemical uptake and directs their site-targeted distribution by genetically modifying plant membrane transporters [2]. However, the difficult part in transporter research is that it undergoes modifications such as phosphorylation and that it has a higher-order structure such as a heterotetramer. In addition, direction of transporter (inword or outword) and localization of transporter in plant is very important [3,4,5]. Most of the information on the physiological roles of these transporters was obtained from studies carried out in model plants species (e.g., *Arabidopsis thaliana* and *Oryza sativa* L.) by screening mutants following genetic mapping, which is a time-consuming and laborious processes. Other in vivo heterologous expression systems, such as *Xenopus* oocytes and yeast, have been effectively used as tools for the functional characterization of these types of transporter proteins.

Several plant membrane proteins have been expressed in oocytes, and their affinity for substrates has been measured by the two-electrode voltage clamp technique [6]. *Xenopus* oocytes are a heterologous expression system that is widely used in biological and pharmacological studies. As a transient expression system, the *Xenopus* oocytes expression system has the advantages of rapid stability and comparative advantages in the understanding of regulatory mechanism on channel proteins [7]. However, the differences between oocytes and plant cells in the physiological environment and resting potential will cause background currents and strong interference signals [8]. In addition, due to codon usage, sometimes transporters are expressed in oocytes at low levels, which make them difficult to be functionally characterized [9]. In addition to oocytes, yeast is a predominant cellular system for heterologous expression of plant membrane proteins. The yeast expression system has the functions of processing and modification after transcription, which makes it suitable for stable expression of plant transporters. Owing to the advantages of both transient and stable expression systems, these systems have been increasingly applied in the field of gene engineering. Most proteins in plants can be expressed in yeast for functional characterization in a high-throughput manner. Moreover, some genes in yeast share high homology with plants, and thus the functions of exogenous genes in plants can be studied by engineering mutants in yeast. However, some restrictions associated with yeast systems have been noted. For instance, not all plant genes are correctly expressed in yeast because of codon usage.

*Nicotiana benthamiana* has served as a model species because of its amenability to transformation and its innate ability to support high levels of heterologous gene expression. *Agrobacterium*-mediated transient expression is a fast method to analyze gene expression; this method is simple and fast, has a short period and results in a high expression efficiency [10,11]. *Agrobacterium*-mediated transient expression in *N. benthamiana* has been widely used to study plant–pathogen interactions, micro–molecular interactions and other processes [12,13]. Transient expression systems with leaf agroinfiltration have shown stable transformation and expression [14]. Since a simple and convenient research tool for the functional characterization of agrochemical transporters is still lacking, we harnessed *Agrobacterium*-mediated transient expression in an *N. benthamiana* system to show its potential in explaining the interaction between xenobiotics and membrane proteins. We established the system by testing the function of tobacco nicotine uptake permease (*NtNUP1*) in nicotine uptake, which has been reported in yeast [15]. Then, the feasibility of the system was further tested by expressing rice polyamine uptake transporter 1 (*OsPUT1*), a gene encoding a protein for which we identified a function in methomyl uptake in a yeast library screen [16]. The results indicated this protocol for analyzing function of plant agrochemical transporter-encoding genes is feasible.

## 2. Results

### 2.1. NtNUP1 Increases Nicotine Uptake in N. benthamiana Foliar Discs

**Figure 1:**
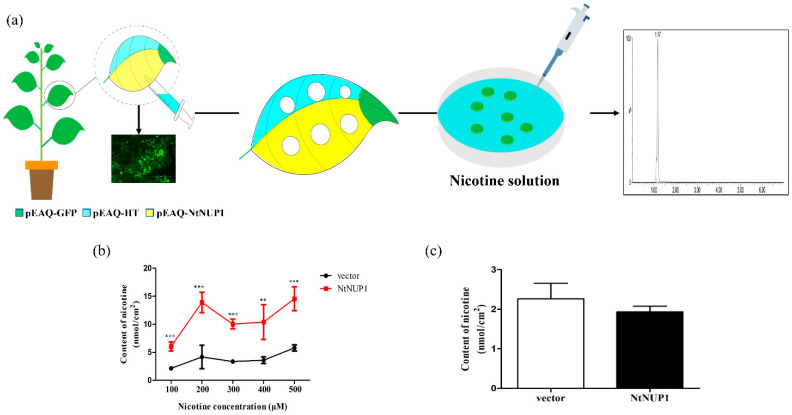
Uptake of nicotine by *N. benthamiana* foliar discs expressing *NtNUP1*. (**a**) Schematic diagram of the nicotine absorption test in *N. benthamiana* foliar disks. After transient expression of pEAQ-*NtNUP1* in *N. benthamiana* for 2 days, 200 mL of 200 μM nicotine was evenly applied to the leaf area where pEAQ-HT and pEAQ-*NtNUP1* were injected. After 24 h, discs (1.54 cm^2^ surface) were obtained from the uniformly illuminated leaf area by the use of a 1.4 cm diameter cork. Upon incubation, the discs were rinsed with a 0.5 mM CaCl_2_ solution three times to remove the remaining nicotine. The final extracted solution was analyzed using liquid chromatography–tandem mass spectrometry (LC-MS/MS). (**b**) Uptake of nicotine by foliar discs at different concentration. Line in red is leaves expressing the *NtNUP1* gene, and that in black is the empty vector control. The data are presented as the means ± standard deviations (*n* = 5) (*** *p* ≤ 0.001; ** *p* ≤ 0.01). (**c**) The content of endogenous nicotine in foliar discs; foliar discs were subjected to direct nicotine detection 24 h after pEAQ-HT and pEAQ-*NtNUP1* were injected.

GFP fluorescence was distributed throughout *N. benthamiana* cells of foliar discs injected with a pEAQ-GFP construct, indicating that tobacco leaves were healthy enough to express foreign genes (Figure 1a). Foliar discs expressing *NtNUP1* were selected to absorb 200 μM nicotine, and their nicotine contents were determined. The nicotine content of the pEAQ-*NtNUP1*-transformed foliar discs was 13.56 nmol/cm^2^, whereas that of the control pEAQ-HT-transformed foliar discs was only 4.22 nmol/cm^2^. The nicotine content absorbed by the pEAQ-*NtNUP1*-transformed foliar discs was significantly higher than that absorbed by the control foliar discs, and this trend was maintained at different nicotine concentrations (100–500 μM), which clearly indicated that *NtNUP1* functions in the uptake of nicotine into leaf cells (Figure 1b).

After pEAQ-*NtNUP1* was transiently expressed, the content of endogenous nicotine in the foliar discs was also determined to eliminate the possibility that the increase in nicotine uptake was due to the aggregation of endogenous nicotine derived from other parts of the plants. The content of endogenous nicotine in the foliar discs injected with pEAQ-*NtNUP1* did not change significantly compared with that in the foliar discs injected with pEAQ-HT (Figure 1c). These results further indicated that the increase in the nicotine content in leaf discs originated from the uptake of the nicotine solution and not from the transfer of endogenous nicotine from other parts of the plants. In summary, *NtNUP1* is involved in concentration-dependent nicotine uptake in foliar discs at low concentrations (100–200 μM). Thus, this *N. benthamiana* transient expression system can be used to explore the xenobiotic uptake function of transporter-encoding genes.

### 2.2. NtNUP1 Increases Nicotine Absorption in N. benthamiana Leaf Protoplasts

**Figure 2:**
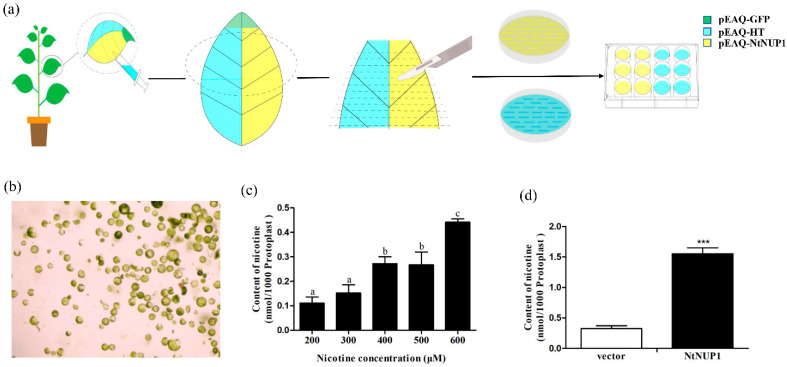
Uptake of nicotine by *N. benthamiana* leaf protoplasts expressing *NtNUP1*. (**a**) Schematic diagram of the nicotine absorption test in *N. benthamiana* leaf protoplasts expressing *NtNUP1.* After the transient expression of pEAQ-*NtNUP1* and pEAQ-HT in *N. benthamiana* for 2 days, leaf protoplasts were isolated. The protoplast suspension and nicotine were transferred to a tissue culture plate. Ten microliters of the supernatant were analyzed via LC-MS/MS. (**b**) The *N. benthamiana* leaf protoplasts prepared from leaves injected with different constructs. (**c**) Uptake of different concentrations of nicotine in untransformed wild-type leaf protoplasts. (**d**) Uptake of 500 μM nicotine by leaf protoplast expressing the pEAQ-HT control and pEAQ-*NtNUP1*. The data are presented as the means ± standard deviations (*n* = 3) (*** *p* ≤ 0.001, the different letters mean significant differences).

We measured the nicotine uptake ability of NtNUP1 in protoplast isolates from leaves injected with pEAQ-*NtNUP1* to further test whether the tobacco transient expression system is suitable for exploring transporter function in protoplasts (Figure 2a,b). On the basis of the results of the uptake of a concentration gradient of nicotine (200–600 μM) in protoplasts derived from wild-type leaves, 500 μM nicotine was used in subsequent experiments (Figure 2c). The nicotine concentration in tobacco leaf protoplasts expressing NtNUP1 was significantly higher than that in the tobacco leaf protoplasts injected with pEAQ-HT (Figure 2d), indicating that *NtNUP1* significantly increased the uptake of nicotine in tobacco leaf protoplasts and that this *N. benthamiana* transient expression system can be used to explore the xenobiotic uptake function of transporter-encoding genes at the tissue and cellular levels.

### 2.3. Uptake of Neonicotinoid Pesticides by N. benthamiana Foliar Discs

**Figure 3:**
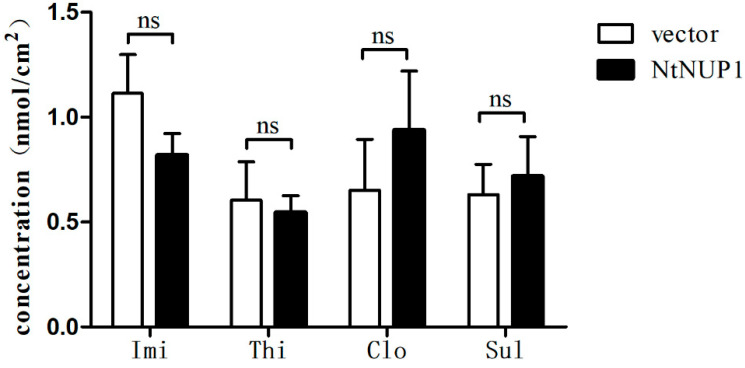
Uptake of neonicotinoid pesticides by *N. benthamiana* foliar discs. The data are presented as the means ± standard deviations (*n* = 5), and ns means no significant difference. The abbreviations lmi, Thi, Clo and Sul represent imidacloprid, thiamethoxam, clothianidin and sulfoxaflor, respectively.

Since some similarities in the structures of neonicotinoid pesticides and natural nicotine have been observed, we further investigated whether *NtNUP1* has the ability to transport neonicotinoid pesticides. Foliar discs expressing *NtNUP1* were treated with 200 μM imidacloprid, thiamethoxam, clothianidin, or sulfoxaflor. The results did not reveal a significant difference in the content of neonicotinoid pesticides between tobacco foliar discs expressing *NtNUP1* and the control group (Figure 3). Thus, *NtNUP1* does not have the function of transporting neonicotinoid pesticides.

### 2.4. Identification of OsPUT1 Methomyl Transporters in Both Yeast Cells and Tobacco Foliar Discs

The function of the *OsPUT1* methomyl transporter was determined both in yeast cells and in the tobacco transient expression transporter system to investigate whether the tobacco transient expression system is feasible for studying other transporters.

**Figure 4:**
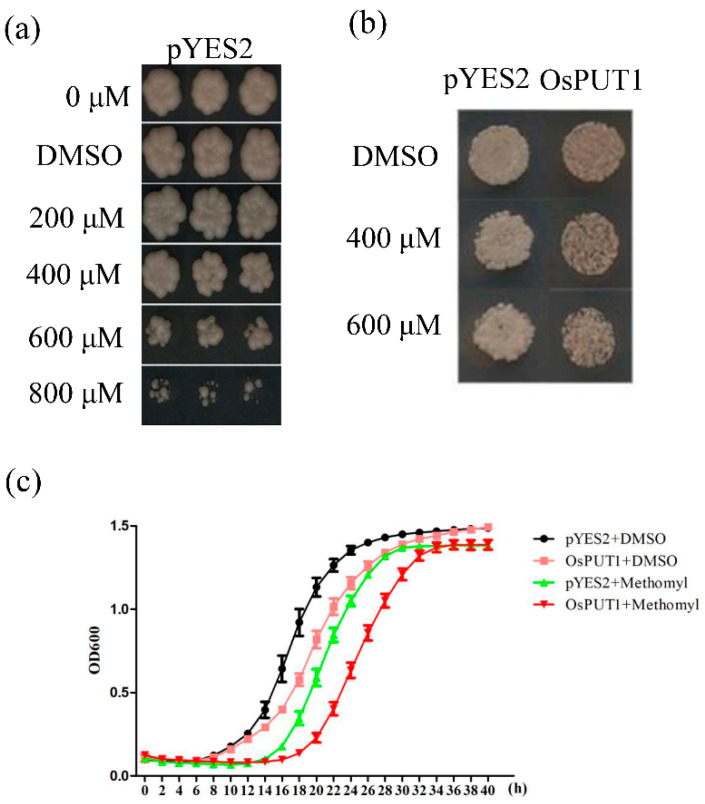
Yeast screens in the presence of different concentrations of methomyl and growth inhibition curves. (**a**) Growth inhibition of pYES2-empty-transformed yeast in media that included different concentrations of methomyl. (**b**) Growth inhibition of yeast expressing pYES2 and *OsPUT1* in the presence of 400 μM and 600 μM methomyl. (**c**) Growth of pYES2- and *OsPUT1*-transformed yeast in synthetic dropout liquid media supplemented with 2% galactose (SD-gal) containing 600 μM methomyl; media that included dimethyl sulfoxide (DMSO) served as a control. The OD_600_ of the culture was recorded every 2 h for 40 h. The data are presented as the means ± standard deviations (*n* = 5).

Based on the sensitivity of yeast to methomyl, the following screening concentrations of methomyl were selected: 400 μM (slight inhibition) and 600 μM (severe inhibition) (Figure 4a). The methomyl transporter-encoding gene *OsPUT1* was screened by using a yeast library enriched in rice transporters [17]. Since methomyl is toxic to yeast cells, we postulated that the growth of yeast cells expressing *OsPUT1* would be inhibited more severely than that of the control strain, due to the uptake of more of the compound into the cells of the former. Upon treatment with 400 μM and 600 μM methomyl, yeast strains expressing *OsPUT1* were more sensitive to methomyl than the wild-type strains (Figure 4b). After 36 h, the growth of yeast expressing *OsPUT1* and pYES2 in each medium reached saturation and remained unchanged (Figure 4c). Overall, *OsPUT1* can increase the uptake of methomyl into yeast cells.

**Figure 5:**
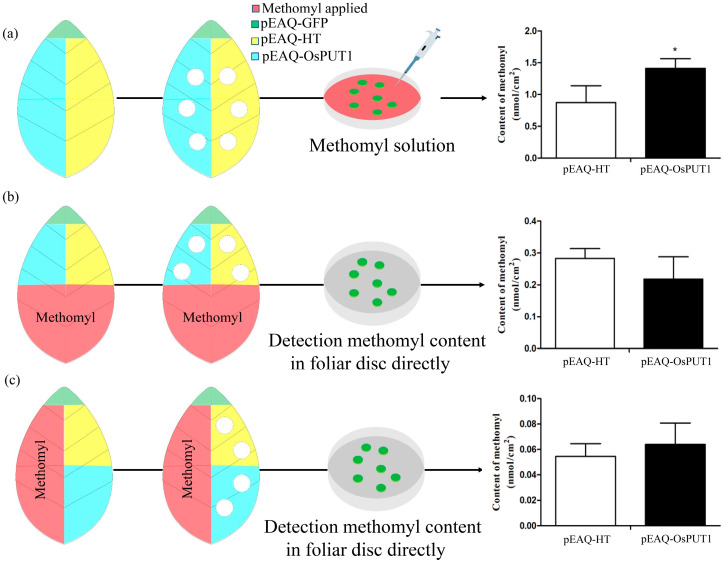
Uptake of methomyl by *N. benthamiana* foliar discs expressing *OsPUT1* after methomyl application to leaves by the use of different methods. (**a**) Methomyl content in foliar discs expressing pEAQ-HT and *OsPUT1.* Foliar discs were immersed in a methomyl solution to measure uptake. (**b**) Content of methomyl in foliar discs expressing pEAQ-HT and *OsPUT1*; methomyl was applied to the top half of leaves and foliar discs from down half were subjected to methomyl detection directly. (**c**) Content of methomyl in foliar discs expressing pEAQ-HT and *OsPUT1;* methomyl was applied to the right side of leaves and foliar discs from left side were subjected to methomyl detection directly. The data are presented as the means ± standard deviations (*n* = 5) (* *p* ≤ 0.05).

Both the uptake and transport of methomyl by *OsPUT1* transporters were verified in the tobacco transient expression transporter system. Tobacco foliar discs successfully expressing *OsPUT1* were measured for their ability to absorb 300 μM methomyl. The content of methomyl absorbed by the pEAQ-*OsPUT1*-transformed foliar discs was 1.41 nmol/cm^2^, and the content of methomyl absorbed by pEAQ-HT-transformed foliar discs was 0.87 nmol/cm^2^ (Figure 5a,b), revealing that the content of methomyl absorbed by pEAQ-*OsPUT1*-transformed foliar discs was significantly higher than the content of methomyl absorbed by the pEAQ-HT-transformed foliar discs.

Two hundred microliters of 300 μM methomyl were applied to the parts of tobacco leaves that were not injected with any vectors to investigate whether *OsPUT1* is involved in translocating methomyl to different leaf positions. Then, the content of methomyl was measured in the foliar discs injected with the pEAQ-HT vector and pEAQ-GFP vector 24 h later. The results indicated that the content of methomyl that translocated to the other side of tobacco did not significantly differ between pEAQ-HT- and pEAQ-*OsPUT1-*transformed leaves (Figure 5c,d) after methomyl was applied to the top half or the right side of tobacco leaves (Figure 5a). These results confirmed that *OsPUT1* mediates the uptake of methomyl and that the tobacco transient expression system is feasible for studying functional xenobiotic transporters *in planta*.

## 3. Discussion

*NtNUP1* increases nicotine absorption in *N. benthamiana* foliar discs and protoplasts, indicating that this transient gene expression system is feasible for studying gene function. The function of proteins in the absorption of pesticides at the plant tissue and cell levels can be verified through different approaches. Although neonicotinoid pesticides originate from natural nicotine, the *NtNUP1* transporter does not have a significant role in the uptake of neonicotinoid pesticides. Therefore, other methods must be used to screen and identify transporters of neonicotinoid pesticides or screen a wider range of pesticide concentrations.

Although we had obtained the methomyl transporter *OsPUT1* by screening a yeast library enriched in rice transporters in a high-throughput way, detailed information about its function in methomyl uptake, translocation and redistribution in the plant must be clarified. Using the tobacco transient expression system, we found that *OsPUT1* increases methomyl uptake by *N. benthamiana* foliar discs and protoplasts, but lateral or longitudinal transfer of methomyl is independent of this transporter. Our results support that *OsPUT1* can uptake methomyl into plants. Notwithstanding, further investigation is needed to test whether other methomyl transporters can transfer it laterally or longitudinally in plants. In addition, to assess critical factors in an agricultural system in the field, such as uptake of the metabolite in specific tissues and/or in cells expressing the transporter, will require stable expression of candidate gene in transgenic plants. The results of the heterologous expression of *OsPUT1* in yeast and tobacco were mutually verified, which further indicated that this transient gene expression system improves the efficiency of analyzing the function of plant genes encoding heterologous transporters. Since transporters are grouped into gene families within plant genomes, modifying this expression system to provide a high-throughput analysis would improve its universality. Two problems must be overcome: one is to clone the CDS region of transporters into a plant expression vector in a high-throughput way and the other is to detect chemicals in a visible manner and in situ. The former difficulty can be overcome by employing homologous recombination mediated by various recombinases [18]. The latter limitation might be overcome by the development of methods such as direct surface analysis coupled to high-resolution mass spectrometry [19,20]. In addition, no transporters have been reported to transport pesticides in the leaves or plants when pesticides are applied to different parts of leaves. The next step is to identify appropriate pesticides and transporter genes to verify the use of this system. Finally, the ability of *OsPUT1* to take up and transport methomyl, as well as the underlying mechanisms, needs further investigation via gene knockout and the use of *OsPUT1*-overexpressing rice plants.

## 4. Materials and Methods

### 4.1. Plant Growth Conditions

*N. benthamiana* plants were cultivated in small pots containing a horticultural potting mixture in a plant growth chamber (XTS408-CC380TLZH, Xutemp, Hangzhou, Zhejiang, China) maintained at 24–26 °C and 65% humidity; 300 μmol/m^2^/s illumination was provided under a 12 h light/12 h dark photoperiod in the artificial climate box.

### 4.2. Chemicals

Nicotine (CAS# 1463371) was purchased from Sigma (Aldrich-Sigma, Burlington, MA, USA), and the other chemicals used in this study were obtained from Cool Lab Biotechnology Co., Ltd., Beijing, China.

### 4.3. Plasmid Construction and Agroinfiltration of N. benthamiana Leaves

The open reading frames (ORFs) of *NtNUP1* and *OsPUT1* were synthesized by Beijing Genomics Institute (Beijing, China) according to the sequences deposited in the GenBank database (accession nos. GU174267 and AK068055, respectively) [15,16]. The ORF for each gene was amplified by primers and cloned into the tobacco leaf transient expression vector pEAQ-HT [21] via an In-Fusion Cloning Kit (Clontech, Mountain View, CA, USA). The resulting plasmids were confirmed by sequencing and maintained in *Agrobacterium tumefaciens* strain LBA4404 [22]. Transient expression of *NtNUP1* and *OsPUT1* was carried out via the agroinfiltration method as described previously [23]. Briefly, cell cultures were expanded to the stable stage in Luria-Bertani media supplemented with 50 µg/mL kanamycin and 50 µg/mL rifamycin, and then pelleted by centrifugation at 2000× *g*. Following resuspension in 10 mM MMA (4-morpholineethanesulfonic acid (MES) (pH 5.8), 10 mM MgCl_2_ and 100 µM acetosyringone) [24] to an OD_600_ of 0.3 and 2–4 h of incubation at room temperature, the suspensions were pressure infiltrated into *N. benthamiana* leaves by the use of a blunt-tipped plastic syringe [25,26]. The top three leaves of three independent plantlets were separately used. Cultures harboring an empty pEAQ-HT vector and pEAQ-GFP vectors were prepared as appropriate, and plants were transferred back to the growth chamber for one day in the dark and one day under the growth conditions mentioned above [27].

### 4.4. Uptake of Chemicals by N. benthamiana Foliar Discs

As described above, the discs (1.54 cm^2^ surface) were obtained utilizing a 1.4 cm diameter cork and pre-incubated for 30 min in foliar discs uptake buffer (20 mM MES (pH = 5.8), 250 mM mannitol, 0.25 mM MgCl_2_ and 0.5 mM CaCl_2_) [28]. In this experiment, 6 discs were used for 1 replicate, with 3 replicates for each concentration, and the discs were incubated with the appropriate concentrations of chemicals (such as nicotine or neonicotinoid pesticides) at 25 °C for 2 h. After the incubation, the discs were rinsed with 50 mL of a 0.5 mM CaCl_2_ solution three times to remove the remaining nicotine. Six milliliters of sterile water were added to the leaf powder after the discs were ground in liquid nitrogen. The homogenate was then ultrasonicated for 30 min and centrifuged at 1699× *g* for 5 min. Afterwards, 1 mL of the supernatant was filtered with a 0.22 µM microporous filter. The final extract solution was subjected to LC-MS/MS or ultra-high performance liquid chromatography (UPLC). For the UPLC determination of neonicotinoid pesticides, the extracts were analyzed using an Agilent 1290 UPLC system (Agilent Technologies, Waldbronn, Germany) attached to an InfinityLab Poroshell 120 SB-C18 chromatographic column (3.0 × 150 mm, 2.7 μm). The solvent was a mixture of acetonitrile and sterile water (*v*/*v*). The injection volume was 5 μL, the flow rate was 0.3 mL/min and the detection wavelength was 234 nm.

### 4.5. N. benthamiana Leaf Protoplast Isolation

After transient expression of pEAQ-*NtNUP1* in *N. benthamiana* for 2 days, the GFP signal in the leaves was observed under an EVOS XL Core microscope (Invitrogen, Carlsbad, CA, USA) [29]. Part of the tobacco leaves injected with pEAQ-HT and pEAQ-*NtNUP1* were cut into thin, approximately 1 mm strips with a blade, and protoplasts were prepared using the methods reported by Chen et al. [2]. After CPW_10_ enzyme (Yakult Pharmaceutical Industry Co., Ltd., Tokyo, Japan) digestion for 1 h at 25 °C, the leaves were placed in 10 mL of enzymatic hydrolysate and hydrolyzed in a 40 rpm shaker for 4 h. The protoplasts and enzymatic hydrolysate were filtered into a 10 mL centrifuge tube and then centrifuged at 100× *g* for 7 min. The precipitate was subsequently suspended in 6 mL of CPW_20_ solution and centrifuged at 100× *g* for 6 min. The protoplasts in the upper suspended liquid were removed and washed with CPW_10_ solution. After centrifugation at 100× *g* for 6 min, the precipitate was collected and rinsed twice; then, the protoplast suspensions were stored on ice for subsequent use.

### 4.6. Uptake of Nicotine by N. benthamiana Leaf Protoplasts

The protoplast suspension and nicotine (diluted with CPW_10_ solution) were transferred to a tissue culture plate. One milliliter of the protoplast and 1 mL of the nicotine solution were added to each well and cultured in the dark at 25 °C for 1 h. The samples from 3 wells were combined into 1 replicate, each of which was repeated three times. The samples were collected into a 10 mL centrifuge tube and centrifuged at 800× *g* for 6 min, after which the supernatant was removed. Three milliliters of CPW_10_ solution were added to resuspend and wash the protoplasts, which were further centrifuged at 800× *g* for 6 min. After repeating the rinsing step twice, 4 mL of CPW_10_ solution were added. After counting with a blood cell counting chamber, we then centrifuged the solution at 800× *g* for 6 min, and the supernatant was removed. The precipitate was then vortexed for 2 min, ultrasonicated for 30 min and centrifuged at 4000× *g* for 5 min. The precipitate and 200 μL of ultrapure water were then mixed together, ultrasonicated for 30 min and centrifuged at 4000× *g* for 5 min. The supernatant was subsequently filtered into a 1.5 mL centrifuge tube with 0.22 μM microporous filter and then centrifuged at 4000× *g* for 10 min. Finally, 10 μL of the supernatant were analyzed using an Agilent 6430 LC/MS system (Agilent Technologies, Santa Clara, CA, USA). The solvents used were acetonitrile (mobile phase A) and aqueous formic acid (mobile phase C). The injection volume was 5 μL, and the flow rate was 0.2 mL per min. The detection procedure was as follows: 0–2.5 min, mobile phase C maintained at 10%; 2.5–3.5 min, mobile phase C changed to 90%; 3.5–5 min, mobile phase C maintained 90%; 5–5.2 min, mobile phase C changed to 10%; and 5.2–7 min, mobile phase C maintained 10%. The mass spectrometry conditions were as follows: retention time, 1.2 min; ionization mode, ESI positive ion mode; capillary voltage, 3 kV; taper hole voltage, 30 V; ion source temperature, 150 °C; desolvent temperature, 600 °C; gas flow rate of the desolvent, 1000 L/h; quantitative ion ratio, 163.1/130.1; qualitative ion ratio, 163.1/130.1; and collision energy, 20 V.

### 4.7. Determination of the Mobility of Methomyl

Three experiments were performed to determine the function of *OsPUT1* and the mobility of methomyl. Experiment 1: After the transient expression of pEAQ-*OsPUT1* in *N. benthamiana* for 2 days, 200 mL of 300 μM methomyl were evenly applied to the leaf area where pEAQ-HT and pEAQ-*OsPUT1* was injected. Experiment 2: After the transient expression of pEAQ-*OsPUT1* in *N. benthamiana* for 2 days, 200 mL of 300 μM methomyl were evenly applied to the top half of leaves. Experiment 3: After the transient expression of pEAQ-*OsPUT1* in *N. benthamiana* for 2 days, 200 mL of 300 μM methomyl were evenly applied to the right side of leaves. After 24 h, discs (1.54 cm^2^ surface) were obtained from the uniformly illuminated leaf area utilizing a 1.4 cm diameter cork. After the incubation, the discs were rinsed with a 0.5 mM CaCl_2_ solution three times to remove the remaining methomyl. Milliliters of sterile water were then added to the leaf powder after the discs were ground into powder in liquid nitrogen. The homogenate was subsequently ultrasonicated for 30 min and centrifuged at 14,000× *g* for 5 min, after which 1 mL of the supernatant was filtered with a 0.22 μM microporous filter. The final extract solution was analyzed using LC-MS/MS. The solvents used were acetonitrile (mobile phase A) and aqueous formic acid (mobile phase C). The injection volume was 5 μL, and the flow rate was 0.2 mL per min. The running procedure was as follows: 0–1.5 min, mobile phase C maintained at 30%; 1.5–4 min, mobile phase C changed to 90%; 4–6 min, mobile phase C maintained at 90%; 6–6.1 min mobile phase C changed to 30%; and 6.1–8 min, mobile phase C maintained at 30%. The mass spectrometry conditions were as follows: retention time, 1.7 min; ionization mode, ESI positive ion mode; capillary voltage, 2.5 kV; taper hole voltage, 15 V; ion source temperature, 150 °C; desolvent temperature, 600 °C; gas flow rate of the desolvent, 1000 L/h; quantitative ion ratio, 163/88 (collision energy, 13 V); and qualitative ion, 163/106 (collision energy, 10 V).

### 4.8. Verification of the Function of OsPUT1 in Methomyl Uptake in Yeast

The pYES2-*OsPUT1* and pYES2 plasmids were introduced into yeast strain W303–1A. We subsequently grew the *OsPUT1*-transformed yeast strains and pYES2-transformed yeast strains (the control group), which were selected from a yeast library, in synthetic dropout liquid media supplemented with 2% glucose (SD-glu) (1.34 g dissolved in YNB, 0.364 g of DO supplement-Ura and 4 g of glucose in 200 mL of water (pH adjusted to 5.8 with NaOH), sterilized at 121 °C for 15 min) (30 °C and shaking at 200 rpm, OD_600_ = 1.0). The yeast were added to 5 mL of SD-gal liquid media that included methomyl (OD_600_ = 0.05) [30]. Then, the yeast (OD_600_ = 0.05) were transferred to a 96-well plate to generate a cell growth curve. The growth during 0–40 h was determined, and the OD_600_ of yeast was measured every 2 h. Each experiment was performed three times, and the mean value of the three replicates was calculated with growth in untreated methomyl media serving as a control.

### 4.9. Statistical Analysis

Statistical analyses were conducted using IBM SPSS software 20 for Windows (https://www.ibm.com/analytics/us/en/technology/spss/, accessed on 20 October 2020), including one-way ANOVA followed by Dunnett’s post hoc test. Graphs were constructed using GraphPad Prism 5 software (https://www.graphpad.com/scientific-software/prism/, 20 October 2020). All of the data are presented as the mean *n* values ± standard deviations (SD) of at least three independent experiments.
